# EXPLORING VIDEO CHAT FOR SOCIAL ENGAGEMENT IN OLDER ADULTS WITH AND WITHOUT COGNITIVE IMPAIRMENT

**DOI:** 10.1093/geroni/igz038.3391

**Published:** 2019-11-08

**Authors:** Lydia T Nguyen, Qiong Nie, Elizabeth Lydon, Dillon Myers, Alan Gibson, Chantal Kerssens, Raksha A Mudar, Wendy A Rogers

**Affiliations:** 1 University of Illinois at Urbana-Champaign, Champaign, Illinois, United States; 3 OneClick.chat, Philadelphia, Pennsylvania, United States; 5 Georgia Institute of Technology, Atlanta, Georgia, United States

## Abstract

Social engagement technologies have the potential to benefit health and quality of life in older adults with and without mild cognitive impairment (MCI). However, technologies are rarely designed to accommodate the interests, capabilities, and limitations of these populations. In the current study, we focused on examining the potential of video chat to socially engage older adults with and without MCI by providing opportunities to link people with shared interests. Eight cognitively normal older adults (Mage: 73.3 years) and five with MCI (Mage: 70.0 years) completed a four-week experiential field trial using a novel online video chat platform called OneClick. System Usability Scale scores at both pre- and post-assessment revealed that OneClick was easy to use for older adults with and without MCI, however individuals with MCI experienced more technical issues and required additional assistance to use the system. Pre- to post- comparisons of questionnaire data revealed positive changes for the Quality of Life, Friendship/Social Isolation, and Loneliness scales in both groups. Of the 13 participants, five cognitively normal and four individuals with MCI reported that they would be interested in continuing to use the video chat system at home to connect with family and friends or to discuss topics of mutual interests. Overall, all participants enjoyed using the video chat system as a means for social engagement and showed trends for social health and quality of life benefits. This field trial illustrates the potential for video chat to provide social engagement opportunities for older adults with and without cognitive impairment.

